# Editorial: Updates on cardiovascular variability: underlying mechanisms and non-pharmacological therapeutic targets

**DOI:** 10.3389/fcvm.2025.1670996

**Published:** 2025-08-14

**Authors:** Gabriel Dias Rodrigues, Rubens Fazan, Nicola Montano

**Affiliations:** ^1^ Department of Clinical Sciences and Community Health, Dipartimento di Eccellenza 2023–2027, University of Milan, Milan, Italy; ^2^ Department of Physiology and Pharmacology, Fluminense Federal University (UFF), Niterói, Brazil; ^3^ Department of Physiology, School of Medicine of Ribeirao Preto, University of Sao Paulo, Ribeirão Preto, Brazil; ^4^ Department of Internal Medicine, Fondazione IRCCS Ca’ Granda, Ospedale Maggiore Policlinico, Milan, Italy

**Keywords:** autonomic nerovus system, baroreflex sensitivity (BRS), machine learning (ML), microgravity, hypoxia, long covid, menopause

**Editorial on the Research Topic**
Updates on cardiovascular variability: underlying mechanisms and non-pharmacological therapeutic targets

The successful translation of heart rate variability (HRV) applications from bench to bedside is largely rooted in the pioneering studies led by Professor Alberto Malliani and his group (1). In essence, a reduction in HRV oscillatory patterns, or an overall loss of cardiovascular variability, reflects disfunction in the modulation of target organ function and serves as a robust prognostic marker (1). Advances in signal processing along with a growing understanding of brain–body interactions, have uncovered new connections between the autonomic nervous system and other biological systems thereby broadening the scope of HRV research.

This Research Topic brings together multidisciplinary contributions that advance our understanding of cardiovascular variability across experimental models, novel methodologies, and clinical applications. Our aim was to provide updated perspectives that bridge from basic physiological mechanisms and applied clinical research, ultimately fostering improved diagnostics, risk stratification, and therapeutic monitoring through the lens of cardiovascular variability assessment.

The studies included in this collection highlight recent advances in the application of cardiovascular variability assessment across a range of clinical and physiological contexts. Scatà et al. reported that postmenopausal women exibit significant reductions in HRV and baroreflex sensitivity compared to age-matched premenopausal controls, pointing to estrogen deficiency in the disruption of autonomic cardiovascular regulation (2). In another study, individuals with long COVID who reported persistent palpitations exhibited altered HRV and baroreflex sensitivity, suggesting that cardiac autonomic imbalance may contribute to the post-acute sequelae of SARS-CoV-2 infection (3).

Dos Santos and colleagues (4) demonstrated that HRV indices combined with machine learning techniques can effectively detect both the presence and severity of obstructive sleep apnea, supporting the development of rapid, non-invasive diagnostic tools. Also, in the field of sleep medicine, multifactorial analysis of HRV revealed altered complexity and scaling behavior in healthy pregnant women compared to non-pregnant controls, offering a sensitive method in capturing subtle autonomic changes during gestation (5).

Beyond clinical aspects, environmental and physiological stressors also modulate cardiovascular variability. Oliveira and colleagues (6) showed that men and women exhibit distinct autonomic responses to hypoxia: women preserved vagal activity and maintained stable baroreflex function, while men exhibited marked reductions in HRV, suggesting sex-specific autonomic adaptations. In the scenario of space medicine, a short-arm human centrifugation protocol delivering artificial gravity mitigated the decline in baroreflex function and HRV typically observed during prolonged bed rest, highlighting its potential as a countermeasure against cardiovascular deconditioning in microgravity simulations (7).

Together, these studies underscore the multifaceted relevance of cardiovascular variability as both a physiological marker and a clinical tool. They highlight the interplay between the autonomic nervous system, clinical features, and the environment influences. As new technologies and analytical frameworks continue to evolve, variability-based metrics are poised to play an increasingly prominent role in personalized medicine, enabling more precise diagnostics and targeted interventions. We hope this Research Topic will inspire continued interdisciplinary collaboration and innovation in the study of cardiovascular variability.
